# Tuneable separation of gold by selective precipitation using a simple and recyclable diamide

**DOI:** 10.1038/s41467-021-26563-7

**Published:** 2021-10-29

**Authors:** Luke M. M. Kinsman, Bryne T. Ngwenya, Carole A. Morrison, Jason B. Love

**Affiliations:** 1 EaStCHEM School of Chemistry, University of Edinburgh, Edinburgh, EH9 3FJ UK; 2 School of Geosciences, University of Edinburgh, Edinburgh, EH9 3FE UK

**Keywords:** Inorganic chemistry, Materials chemistry, Supramolecular chemistry

## Abstract

The efficient separation of metals from ores and secondary sources such as electronic waste is necessary to realising circularity in metal supply. Precipitation processes are increasingly popular and are reliant on designing and understanding chemical recognition to achieve selectivity. Here we show that a simple tertiary diamide precipitates gold selectively from aqueous acidic solutions, including from aqua regia solutions of electronic waste. The X-ray crystal structure of the precipitate displays an infinite chain of diamide cations interleaved with tetrachloridoaurate. Gold is released from the precipitate on contact with water, enabling ligand recycling. The diamide is highly selective, with its addition to 29 metals in 2 M HCl resulting in 70% gold uptake and minimal removal of other metals. At 6 M HCl, complete collection of gold, iron, tin, and platinum occurs, demonstrating that adaptable selective metal precipitation is controlled by just one variable. This discovery could be exploited in metal refining and recycling processes due to its tuneable selectivity under different leaching conditions, the avoidance of organic solvents inherent to biphasic extraction, and the straightforward recycling of the precipitant.

## Abstract (web summary)

The separation of metals from electronic waste is an enduring technological and societal challenge, and new metal extraction, refining and recycling solutions are needed. Here the authors report a recyclable and tuneable chemical reagent that separates valuable metals such as gold by direct and selective precipitation from various acidic, mixed-metal solutions of relevance to extraction and recycling industries.

## Introduction

The extraction of gold from its ores and its recycling from waste materials represent significant technological and environmental challenges^1–4^. Alternatives to the current industrial practice of cyanidation^5,6^ and informal mercury alloying^7,8^ that are based upon selective extraction, precipitation, or adsorption of gold from leach solutions are being actively pursued^9^, with the recycling of electronic waste (e-waste) the focus of much attention as it presents a significantly higher concentration of gold than its ores^10^. Recycling these latter materials would also provide impetus to global circular economy visions^11,12^, but their complexity requires highly selective and environmentally benign recycling technologies^13,14^.

Selective and reusable precipitation and adsorption methods are becoming increasingly popular for gold and other metal separations as they provide significant advantages over traditional, single-use precipitants^15–19^. Pre-formed porous network materials such as metal-organic frameworks (MOFs), polymers, and electroactive materials are selective for gold adsorption, and often exploit the accessible reduction potential of gold to deposit the metal within the porous cavities^20–25^. Molecular recognition processes involving cyclodextrins^26,27^ and cucurbit[*n*]urils (*n* = 5–8)^28–30^ use the curvature and donor-group decoration of these capsular guest molecules to host selectively both alkali-metal cations and AuCl_4_^−^ within superstructures that ultimately precipitate gold from aqueous acidic solutions. This rationale has also been extended to the separation by precipitation of platinum from palladium and rhodium in which the cucurbituril displays a preference for the hexachloroplatinate(IV) dianion^31^. Supramolecular interactions between acyclic durene diamides and HAuCl_4_ resulted in gold precipitation as extended networks, although in these cases no selectivity for gold over other metals was explored^32^. Extended supramolecular network structures were also formed upon selective precipitation of HAuCl_4_ from acidic solutions comprising Au, Ni, Cu, Zn, alkali-, and alkaline-earth metals by the biomolecule niacin, a pyridine carboxylic acid^33^.

**Fig. 1:**
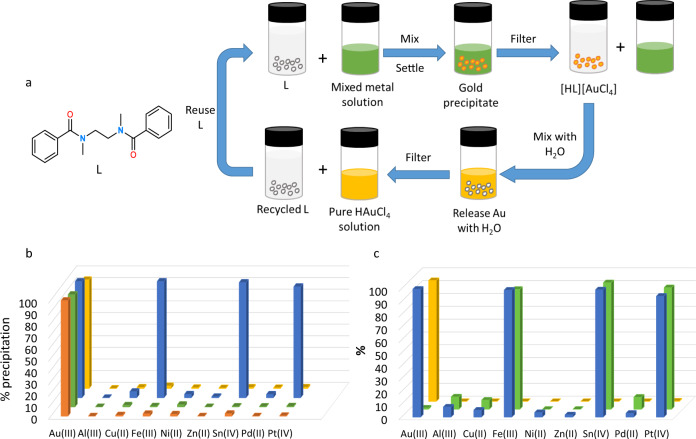
Schematic of the precipitation process and its selectivity. **a** Chemical structure of L and schematic of the precipitation process. **b** Percentage metal(s) removed by precipitation from a 0.01 M mixed-metal solution in 2 M (orange and green bars) or 6 M (blue and yellow bars) HCl following the addition of either 0.2 mmol L (10-fold excess L relative to metal, orange and blue bars) or 0.02 mmol L (equimolar, green and yellow bars). **c** Selective metal precipitation and stripping sequence. Blue bars: Percentage metal removed by precipitation from a 0.01 M mixed-metal solution in 6 M HCl. Green bars: percentage of metal stripped from the precipitate by a 2 M HCl wash. Yellow bars: percentage of metal stripped from the precipitate after a subsequent wash with deionised water. Experiments carried out in triplicate.

We reported that simple monoamides showed high selectivity in the separation of gold by solvent extraction (SX) from acidic solutions of metals representative of e-waste^34,35^. Protonation of the monoamides formed intermolecular proton-chelated receptor cations in the organic phase that dynamically assembled with AuCl_4_^−^ into supramolecular clusters. In some cases, mixed-metal third phases formed, along with a tin-containing precipitate^35^. The observation of proton-linked amides by us and others in gold separations suggested that the simple diamide (L) (Fig. 1a) would readily assemble into a proton-chelated structure suitable for metalate recognition along with a potential to form insoluble, infinitely extended structures. A resulting selective precipitation could both eradicate the use of potentially hazardous organic solvents in SX separations and simplify a hydrometallurgical separation process. Here we show that diamide L acts as a highly selective reagent for the precipitation of gold from a variety of aqueous acidic solutions and, by tuning the HCl concentration, allows other valuable and critical metals to be separated by precipitation.

## Results and discussion

### Precipitation experiments

Initial screening of L in SX experiments showed fast gold transport from 2 M and 6 M HCl to form yellow chloroform phases but, as desired and in contrast to previous results^35^, yellow precipitates form that contain all of the gold. The diamide L was therefore evaluated as a precipitant in the absence of the organic solvent (Fig. 1). Adding 0.2 mmol of solid L, the equivalent in moles used in the SX experiments (i.e., a 10-fold excess), to a 0.01 M HAuCl_4_ solution in 2 M or 6 M HCl results in colourless supernatants and yellow solids which incorporate >99% of the gold. Analysis of these supernatants by ICP-MS shows residual gold concentrations of 5.9 ppm and 2.3 ppm in 2 M and 6 M HCl, respectively (Supplementary Table 1). Repeating these experiments using significantly more dilute Au solutions (10 ppm) results in 89% and 84% Au precipitation from 2 M and 6 M HCl solutions, respectively, with residual gold concentrations of approximately 1 ppm in the supernatants. Furthermore, complete gold precipitation using L is also seen from solutions of HAuCl_4_ in 20 or 100% aqua regia or 2 M H_2_SO_4_ in the presence of chloride or bromide (Supplementary Table 1), indicating that the process should be suited for a range of leaching conditions. Moreover, the use of precipitation instead of SX overcomes safety and environmental issues inherent with the use of organic solvents on a large scale. Significantly, the release of gold from the isolated solids is achieved by washing with deionised (DI) water, resulting in transport of HAuCl_4_ into solution and the recycling of L (Supplementary Table 2). The recycled diamide can be used in further gold load/strip cycles showing 87% loading efficacy after three cycles (Supplementary Information, Table 3); this decrease from the initial 99% gold precipitation after three cycles is likely due to sampling errors while handling milligram quantities of the diamide.

### Selectivity of precipitation

The uptake of gold by L from mixtures of metals in HCl is highly selective. The addition of 0.2 mmol of solid L to a mixed-metal solution comprising 0.01 M each of Au, Al, Cu, Ni, Fe, Zn, Pt, Pd, and Sn in 2 M HCl results in near quantitative removal of Au with minimal co-precipitation of other metals (<5%, Fig. 1b, orange bars). It is notable that using stoichiometric L results in gold uptake only (i.e., 0.02 mmol, Fig. 1b, green bars) which contrasts with SX conditions where an excess of extractant is required, thus highlighting the enhanced atom economy of this precipitation method. At 6 M HCl using excess L, complete uptake of Fe, Sn, and Pt is also seen, alongside Au, from the above mixture of metals (Fig. 1b, blue bars), and is likely due to an increased propensity to form the chloridometalates FeCl_4_^−^, SnCl_6_^2−^, and PtCl_6_^2−^ at higher HCl concentrations. Using stoichiometric L, however, a return to selective gold uptake is seen (Fig. 1b, yellow bars), which shows that a process could be designed to sequentially precipitate Au then, depending on the feed stream, Fe, Sn, or Pt. This is significant as leach solutions from gold ores (typically pyrite or arsenopyrite) are rich in iron, while those derived from e-waste have high concentrations of tin^36^. Furthermore, the selectivity shown between Pt(IV) and Pd(II) at 6 M HCl is notable as this separation is integral to precious metal refining processes currently based on SX^37^. The selectivity seen under stoichiometric conditions also suggests that the preference for gold precipitation is not wholly dependent on the ease of formation of HAuCl_4_ compared with other chloridometalates, but that the chemical structures of the precipitates also define the sequence of separation (see structural analysis later).

The selective uptake of Au at 2 M HCl compared with the requirement for 6 M HCl to load Fe, Sn, and Pt permits a selective stripping process to be undertaken. As such, loading L with Au, Fe, Pt, and Sn at 6 M HCl (Fig. 1c, blue bars), followed by a wash with 2 M HCl results in dissolution of Fe, Sn, and Pt only, with Au retained on the solids (Fig. 1c, green bars). Washing the isolated solids with DI water releases the Au into solution and recycles L (Fig. 1c, yellow bars). Reduction of this solution by sodium metabisulfite yields metallic gold of 97.04% purity (Supplementary Table 4).

The selectivity of L for Au uptake was evaluated further by adding an excess to mixed-metal ICP-OES standard solutions (diluted in 2 M or 6 M HCl), comprising 29 metals at 100 or 10 ppm concentrations. Analysis of the concentrations of metals that remain in solution reveals that even in this competitive environment, L is highly selective for gold, with 70% uptake after 24 h (Supplementary Fig. 1). Raising the concentration of HCl to 6 M increases the uptake of Au to >99% but decreases selectivity, with Tl (95%), Ga (>99%), and Fe (70%) also precipitated; however, these metals could in principle be removed from the precipitate by a 2 M HCl wash (see above). Interestingly, no Pt uptake is seen and is due to it being present as Pt(II) (i.e., PtCl_4_^2−^) and not Pt(IV) (i.e., PtCl_6_^2−^), showing that the structure of the chloridometalate is important to the precipitation process.

Finally, gold was selectively separated from end-of-life printed circuit boards dissolved in aqua regia (diluted to 40%), with 98% Au precipitation after 1 h and no co-precipitation of any of the other elements present (Supplementary Table 5 and Supplementary Fig. 2). This contrasts with SX experiments using secondary and tertiary monoamides that formed a dense organic third phase comprising gold, iron, and tin on contact with model e-waste solutions^35^.

### Structural analysis of precipitates

**Fig. 2:**
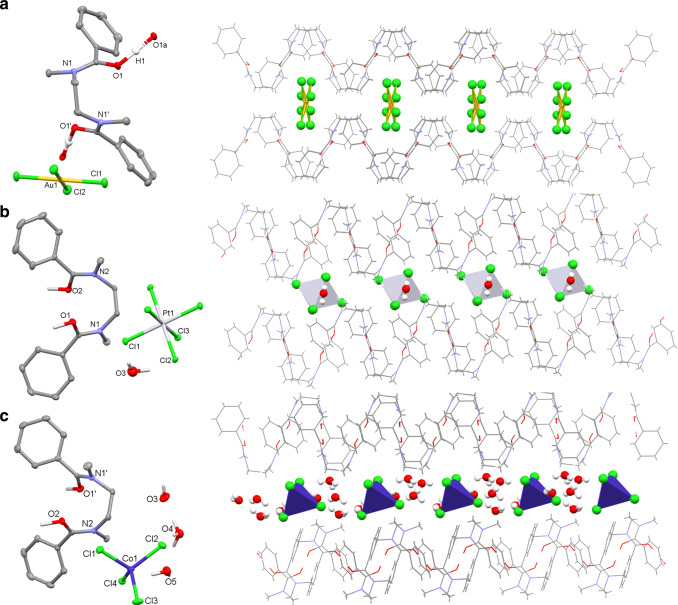
Structural characterisation of precipitates by X-ray crystallography. For clarity, all hydrogens except those involved in hydrogen bonding are omitted (displacement ellipsoids are drawn at 50% probability). Atom colours: red = O; cyan = N; grey = C; green = Cl; yellow = Au, pale grey = Pt, dark blue = Co; white = H. **a** X-ray crystal structure of [HL][AuCl_4_] showing the intermolecular proton-chelate structure and the arrangement of AuCl_4_^−^ within the rhombohedral clefts derived from the phenyl and methyl substituents of the infinite chain of protonated diamides, C(H)—Cl(Au) 3.43–3.97 Å; N1-C3-C3′-N1′ 54.9(3)°. **b** X-ray crystal structure of [HL]_2_[PtCl_6_](H_2_O) showing the arrangement of PtCl_6_^2−^ and a molecule of water within the intermolecular proton-chelated structure. For clarity, only one of the two [HL] cations is shown. **c** X-ray crystal structure of [HL][H_3_O(H_2_O)_2_][CoCl_4_] showing the intermolecular proton-chelate structure and the layered arrangement of CoCl_4_^2−^ and H_3_O^+^ water cluster between the infinite ribbon chain of protonated diamides.

**Fig. 3:**
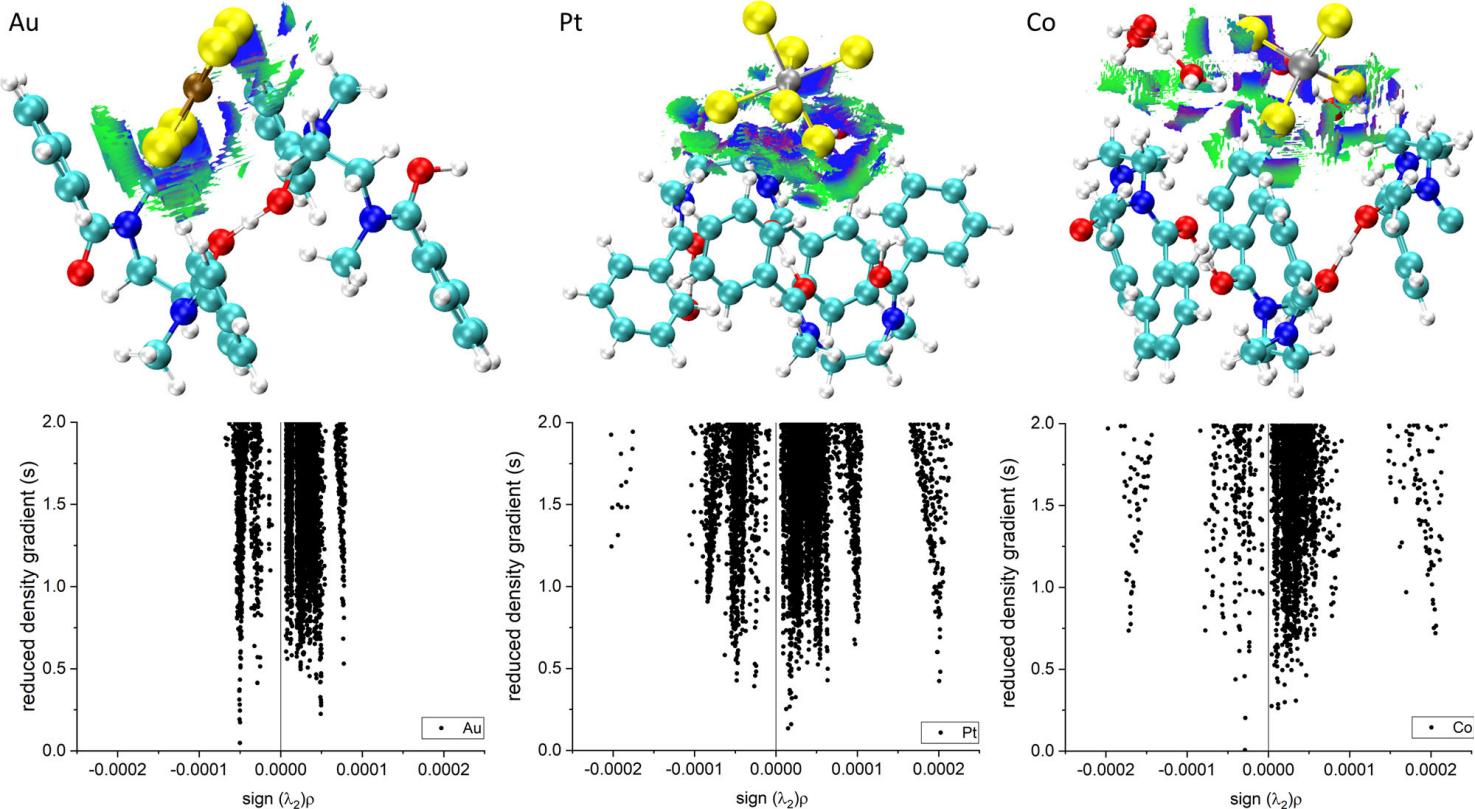
Non-covalent interaction (NCI) analysis. Non-covalent bonding interactions 3D isosurface plots (*s* = 1.0 au, −0.00025 (dark blue scale) < *ρ* < 0 (green scale) au) (top), and 2D *ρ* vs *s* plots (bottom) for the crystal structures of [HL][AuCl_4_], [HL]_2_[PtCl_6_].H_2_O, and [HL][H_3_O(H_2_O)_2_][CoCl_4_]. Atom colours: cyan = C, red = O, dark blue = N; yellow = Cl; white = H; brown = Au; grey = Pt or Co.

Layering a solution of 0.01 M HAuCl_4_ in 2 M HCl on a 0.1 M chloroform solution of L results in controlled crystallisation. The X-ray crystal structure (Fig. 2a) shows a chemical formula of [HL][AuCl_4_] in which the unique proton H1 is bound between adjacent amide O-atoms O1 and O1a (O1—O1a = 2.420(3) Å), forming an intermolecular proton chelate between amide units that assemble into an infinite supramolecular chain motif. While the linking of the diamides in [HL][AuCl_4_] is similar to that seen for HAuCl_4_ complexes of the diamidodurene R’C(O)N(R)CH_2_(C_6_Me_4_)CH_2_N(R)C(O)R’^32^, the positioning of the anions is different. In the latter example, the π-rich aryl group interacts strongly through face-to-face π-bonding with the planar AuCl_4_^−^, whereas for [HL][AuCl_4_] the phenyl and methyl substituents within the ribbon-like structure of the protonated diamides provide rhombohedral clefts that host the AuCl_4_^−^ guest. A non-covalent interaction (NCI) plot (Fig. 3) shows that the ligand clefts provide stabilising van der Waal interactions through the aryl ring and amide linkage, alongside multiple non-classical C-H∙∙∙Cl hydrogen-bonding contacts.

Crystallisations of [HL]_2_[PtCl_6_], [HL][FeCl_4_], and [HL]_2_[SnCl_6_] were also achieved (Fig. 2b and Supplementary Fig. 4a, b, respectively). All three complexes are structurally similar, adopting a ribbon motif of [HL]^+^ with an intermolecular proton chelate analogous to that seen for [HL][AuCl_4_]. This results in a similar rhombohedral cavity which accommodates [FeCl_4_]^−^ but which is offset in the structures of [HL]_2_[SnCl_6_] and [HL]_2_[PtCl_6_], the latter two structures also accommodating one molecule of water per asymmetric unit. The N–C–C–N torsion angle of the diamide bridge varies according to the metalate and is significantly smaller for Au (54.0°) than the other metals (Fe: 81.0/88.2; Sn 80.0; Pt 80.6°). The NCI plots (Fig. 3 and Supplementary Fig. 3) reveal that the nature of the host–guest interactions switches to C-H∙∙∙Cl, with the water molecule in the [HL]_2_[SnCl_6_] and [HL]_2_[PtCl_6_] structures providing additional non-classical hydrogen-bonding to both the ligand and metalate ions. The host/guest interactions in these complexes are of a stronger nature (more negative *ρ*) compared with [HL][AuCl_4_].

Metals such as cobalt and zinc that are not precipitated by L remain in solution. However, at 10 M HCl, a concentration at which L is completely soluble, some crystallisation occurs and the X-ray crystal structures show the formation of [HL][H_3_O(H_2_O)_2_][CoCl_4_] (Fig. 2c) and [HL][H_3_O(H_2_O)_2_][ZnCl_4_] (Supplementary Fig. 4c). In both cases, [HL]^+^ forms an infinite intermolecular proton-chelated motif, with N–C–C–N torsional angles of 72.5 and 72.6° for Co and Zn, respectively. Protonated water clusters [H_3_O(H_2_O)_2_]^+^ are found instead of a second [HL]^+^, resulting in well-defined layers of [H_3_O(H_2_O)_2_]^+^/[MCl_4_]^2−^ interleaved with [HL]^+^. The NCI plots (Fig. 3 and Supplementary Fig. 3) again support C-H∙∙∙Cl host/guest interactions, bridged with non-classical hydrogen-bonding from the protonated water clusters. Thus, it can be concluded that distinct structural differences exist between the complexes that readily precipitate from aqueous acid compared with those that do not. There is also a marked preference for [HL]^+^/metalate ion-pair formation in the solid state, even under the highly biased relative concentrations of metalate (0.01 M) vs. chloride (2–10 M HCl), which has favourable implications for process mass balance.

### Mechanism of precipitation

The discovery that full uptake of gold from solution occurs using a stoichiometric amount of L suggests that a dissolution-precipitation, not a surface-deposition mechanism, is occurring. This is supported by analysis of the rate of gold uptake at various concentrations of HCl (Supplementary Figs. 5 and 6), which is found to be related to the extent of dissolution of L. Addition of HAuCl_4_ to a solution of L in 12 M HCl results in the rapid and wholesale precipitation of [HL][AuCl_4_]. Dissolution of L in 2 M HCl, as determined by quantitative ^1^H NMR spectroscopy, is minimal at 0.6 mM, while heating this solution to 340 K increases the concentration of dissolved L to 0.8 mM (Supplementary Fig. 7, Supplementary Table 6). Increasing the concentration of HCl to 6 M results in a 15-fold increase to 8.7 mM at 300 K (Supplementary Fig. 8, Supplementary Table 7). The increase in dissolved L mirrors the increase in quantity of [HL][AuCl_4_] precipitated from 2 M HCl over 5 min, which doubles on raising the temperature from 20 to 40 °C and from 40 to 80 °C (Supplementary Fig. 9).

The precipitate from the addition of 0.02 mmol of L to a solution of 0.01 M HAuCl_4_ in 6 M HCl was analysed by powder X-ray diffraction (Supplementary Fig. 10) and showed the presence of two phases commensurate with a mixture of microcrystalline [HL][AuCl_4_] and L. Refinement of the unit cell parameters in space group *I*2/*a* (*a* = 12.216(1) Å, *b* = 17.099(5) Å, *c* = 16.721 (3) Å, *β* = 142.79 (1) °) shows a close match with those of the single-crystal X-ray data for [HL][AuCl_4_] (*a* = 12.0848(7) Å, *b* = 16.9574(2) Å, *c* = 16.5644(10) Å, *β* = 142.07(1) °), further supporting a dissolution-precipitation mechanism.

We have shown that a simple diamide acts as a very selective and recyclable precipitant for gold under a variety of conditions relevant to metal separation processes. Metal uptake is tuneable and related to the acidity of the leach solution and the structures of the precipitates, allowing for further tailoring of these compounds to target different metal separations. The use of this highly selective and recyclable precipitation method precludes the use of organic solvents inherent to SX separations and may provide a simple solution towards environmentally benign metal recycling.

## Methods

All solvents and reagents were used as received from Sigma-Aldrich, Fisher Scientific UK, Alfa Aesar, Acros Organics or VWR International. Deionised water was obtained from a MilliQ purification system.

### Synthesis of L

Diamide L was prepared according to a procedure adapted from the literature^38^. Under argon, N,N′-dimethylethylenediamine (1.76 g, 2.15 mL, 20 mmol) was dissolved in CH_2_Cl_2_ (30 mL) and treated with NEt_3_ (7 mL). A solution of benzoyl chloride (7.03 g, 5.81 mL, 50 mmol) in CH_2_Cl_2_ (20 mL) was slowly added and the resulting solution stirred at r.t. for 18 h. The solution was then diluted with CH_2_Cl_2_ (50 mL) and washed with 1 M HCl (3 × 100 mL) and brine (3 × 100 mL). The organic layer was dried over sodium sulfate and evaporated to dryness under vacuum. The resulting solid was recrystalised from hot toluene to yield L as a white powder (4.54 g, 77%).

### Precipitation procedure for 0.01 M mixed metal solutions

Hydrochloric acid solutions (2 M and 6 M) were prepared by dilution of concentrated hydrochloric acid with deionised water. Mixed-metal solutions (0.01 M) were typically prepared by dilution of 0.1 M stock solutions of each individual metal salt solution in 2 or 6 M HCl. The resulting pHs of all solutions were recorded as zero. Solid L (0.2 mmol or 0.02 mmol) was added to a vial with a magnetic stir bar and the metal-containing aqueous solution (2 mL) added. The mixture was stirred for 1 h at room temperature (20 °C) at 500 rpm after which the stir bar was removed and the vial centrifuged. The supernatant was decanted and samples prepared for ICP-OES analysis to measure the uptake of metal by L. Samples were diluted by 100x in 2% nitric acid prior to ICP-OES analysis. This procedure was repeated in triplicate.

### Selective precipitation of gold from 28 other elements procedure

The following ICP multi-element standard solutions were used: Transition metal mix 3 for ICP supplied by Sigma Aldrich comprising 100 mg L^−1^ Au, Ir, Os, Pd, Pt, Rh, Ru in 10% hydrochloric acid and ICP multi-element standard solution IV comprising 1000 mg L^−1^ Ag, Al, B, Ba, Bi, Ca, Cd, Co, Cr, Cu, Fe, Ga, In, K, Li, Mg, Mn, Na, Ni, Pb, Sr, Tl, Zn in dilute nitric acid. Each solution (1 mL) was diluted to 10 mL using either 2 M HCl or 6 M HCl, resulting in solutions of 10 mg L^−1^ Au, Ir, Os, Pd, Pt, Rh, Ru and 100 mg L^−1^ Al, B, Ba, Bi, Ca, Cd, Co, Cr, Cu, Fe, Ga, In, K, Li, Mg, Mn, Na, Ni, Pb, Sr, Tl, Zn. The solutions were filtered prior to use in precipitation experiments due to the precipitation of silver chloride, which was subsequently excluded from ICP-OES analysis. The precipitation method used for the 0.01 M mixed-metal solutions was followed.

### Selective precipitation of gold from waste printed circuit boards

End-of-life printed circuit boards were supplied by Edinburgh School of Chemistry workshop. Gold-tipped sections of the circuit boards (22.85 g) were cut off and soaked in 100 mL aqua regia for 24 h. This solution was then diluted with deionised water to 250 mL and the metal content analysed by ICP-OES. An aliquot of the e-waste solution (2 mL) was stirred with L (0.0059 g, 0.02 mmol, excess with respect to the gold concentration) for 1 h at room temperature after which the stir bar was removed and the vial centrifuged. The supernatant was decanted and samples prepared for ICP-OES analysis to measure the uptake of metal. Samples were diluted by 1000x and 20x in 2% nitric acid prior to ICP-OES analysis. This procedure was repeated in triplicate.

### Crystallisation procedures

[HL][AuCl_4_]: Light yellow prisms were grown at RT from a 0.01 M solution of HAuCl_4_ in 2 M HCl layered on a 0.1 M solution of L in chloroform. [HL][FeCl_4_]: Yellow needles were grown at RT from a 0.01 M solution of FeCl_3_ in 6 M HCl layered on a 0.1 M solution of L in chloroform. [HL]_2_[SnCl_6_](H_2_O): Colourless plates were grown from a 0.01 M solution of SnCl_4_ in 6 M HCl layered on a 0.1 M solution of L in chloroform. [HL]_2_[PtCl_6_](H_2_O): Yellow/orange slabs were grown from a mixture of 0.01 M Na_2_PtCl_6_ and L in 6 M HCl. [HL][H_3_O(H_2_O)_2_][CoCl_4_]: Translucent dark blue plates were grown at RT from a mixture of 0.01 M CoCl_2_ and L in 10 M HCl. [HL][H_3_O(H_2_O)_2_][ZnCl_4_]: Colourless plates were grown at 4 °C from a mixture of 0.01 M ZnCl_2_ and L in 10 M HCl.

### Elemental analysis

Elemental analyses were determined by Elemental Microanalysis Ltd and were measured in duplicate.

[HL][AuCl_4_]: Calculated for C_18_H_21_N_2_O_2_AuCl_4_ (M_r_ = 636.14 g mol^−1^): C, 33.99; H, 3.33; N, 4.40%. Found: C, 34.18; H, 3.32; N, 4.50%.

[HL][FeCl_4_]: Calculated for C_18_H_21_N_2_O_2_FeCl_4_ (M_r_ = 495.02 g mol^−1^): C, 43.67; H, 4.28; N, 5.66%. Found: C, 43.86; H, 4.23; N, 5.80%.

[HL]_2_[SnCl_6_](H_2_O): Calculated for C_36_H_44_N_4_O_5_SnCl_6_ (M_r_ = 944.18 g mol^−1^): C, 45.80; H, 4.70; N, 5.93%. Found: C, 46.31; H, 4.53; N, 6.08%.

[HL]_2_[PtCl_6_](H_2_O)_2_: Calculated for C_36_H_46_N_4_O_6_PtCl_6_ (M_r_ = 1038.57 g mol^−1^): C, 41.63; H, 4.46; N, 5.39%. Found: C, 41.30; H, 4.16; N, 5.60%.

### Timed gold precipitation experiments

Solutions of HAuCl_4_ (0.01 M) were prepared in 2, 4, or 6 M HCl. Solid L (0.02 mmol) was added to a vial with a magnetic stir bar and the relevant aqueous metal solution (2 mL) added. The mixture was stirred for between 1 minute* and 55 min after which the stir bar was removed and the vial centrifuged for 5 min. The supernatant was decanted and samples prepared for ICP-OES analysis to measure the uptake of metal. Samples were diluted by 100x in 2% nitric acid prior to ICP-OES analysis. *One-minute experiments were not centrifuged and instead stirred for 30 s before removing the stir bar and allowing any solids to settle for an additional 30 s. A clear 0.1 mL aliquot was then sampled immediately and prepared for ICP-OES analysis.

### Quantitative NMR solubility experiments

^1^H NMR spectra were recorded on a Bruker Avance III 400 MHz NMR spectrometer. 2 M and 6 M HCl solutions were diluted from concentrated HCl with D_2_O. A theoretical 0.1 M solution of L in 2 M or 6 M HCl was prepared by adding L (0.0178 g) to an NMR tube along with the relevant HCl/D_2_O solution (0.55 mL) and 1 M *tert*-butanol in D_2_O (0.05 mL) as an internal standard. Any undissolved solids were allowed to settle to the bottom of the NMR tube before acquiring ^1^H NMR spectra. ^1^H NMR spectra were acquired for 2 M HCl solutions between 300–340 K in 10 K increments and for 6 M HCl solutions between 300–320 K in 10 K increments; attempts to acquire additional spectra beyond 320 K for these latter samples were unsuccessful due to excessive line broadening of the spectra and difficulties with sample locking.

### Selective stripping experiments with H-tube apparatus

Solid L (0.2 mmol) was added to one side of the H-tube (Supplementary Fig. 11) with a stir bar. The metal-containing aqueous solution (2 mL) was then added to the solids and the mixture stirred for 1 h at room temperature at 500 rpm, after which it was passed through the glass frit of the H-tube with the aid of compressed air or N_2_ gas (See Supplementary Movie 1). The filtrate was collected for ICP-OES analysis to determine metal uptake. The solids were subsequently washed with 2 M HCl (3 × 2 mL) for 30 min, with each 2 mL solution being passed through the glass frit of the H-tube. The solids were then washed with ultrapure deionised water (5 × 2 mL) in the same manner. The use of a H-tube allows for all solids to be retained in the same vessel to minimise any loss of metal due to material transfer. This procedure was repeated in duplicate.

### Purity of recovered gold from 0.01 M mixed metal solution

A solution of HAuCl_4_ in ultrapure deionised water resulting from a load/strip cycle from a 0.01 M mixed-metal solution was reduced by addition of sodium metabisulfite. The resulting solids were filtered and dried. 1.7 mg of solids was re-dissolved in aqua regia (3 mL) and diluted 100x for ICP-OES analysis. The purity of the reduced Au was found to be 97.04%.

### Recycling experiments

A solution of HAuCl_4_ (0.01 M, 2 mL) in 6 M HCl was stirred with L (0.059 g, 0.02 mmol) in a vial for 15 min. The mixture was centrifuged and the supernatant liquors pipetted from the solids, after which ultrapure deionised water (5x2 mL) was added and the mixture stirred for 15 min. The mixture was then centrifuged and the solution pipetted from the solids for each wash cycle. This load/strip procedure was repeated three times.

### ICP-OES analysis

Quantitative metal analysis was carried out on a Perkin Elmer Optima 5300DC Inductively Coupled Plasma Optical Emission Spectrometer. Samples in 2% nitric acid were taken up by a peristaltic pump at a rate of 1.3 mL min^−1^ into a Gem Tip cross-flow nebuliser and a glass cyclonic spray chamber. Argon plasma conditions were 1500 W RF forward power and argon gas flows of 12, 1.0, and 0.6 L min^−1^ for plasma, auxiliary, and nebuliser flow, respectively. ICP-OES calibration standards were obtained from VWR International, Merck Millipore, or Sigma-Aldrich. Selected emission wavelengths are detailed in Supplementary Table 8. Data are rounded to 3 significant figures after incorporating the appropriate dilution factors (typically 100x unless otherwise stated).

### ICP-MS analysis

ICP-MS analysis was carried out by Agilent LTD on an Agilent 7850 Single Quadrupole Inductively Coupled Plasma Mass Spectrometer. Samples in 2% nitric acid were taken up by a peristatic pump at a rate of 0.3 rps into a MicroMist nebuliser and a quartz Scott type spray chamber. Argon plasma conditions were 1550 W RF power and gas flows of 15, 1.07, and 0.9 L min^−1^ for plasma, auxiliary, and nebuliser flow, respectively.

### UV–Vis analysis

UV–Vis spectra of [HL][AuCl_4_] were recorded in acetonitrile in quartz cuvettes (1 cm^3^ path length) on a Shimadzu UV-1900 UV-VIS spectrophotometer (Supplementary Fig. 5). A 1 mM stock solution was prepared by dissolving dry [HL][AuCl_4_] (6.4 mg) in acetonitrile. This solution was then diluted to lower concentrations by serial dilution to obtain measurable UV–Vis spectra.

### X-ray crystallography

X-ray crystallographic data were collected at 100 K or 120 K on an Oxford Diffraction Excalibur diffractometer using graphite monochromated Mo-K_α_ radiation equipped with an Eos CCD detector (*λ* = 0.71073 Å), or at 100 K or 120 K on a Supernova, Dual, Cu at Zero Atlas diffractometer using Cu-K_α_ radiation (*λ* = 1.5418 Å), or at 100 K on a Bruker *APEX*-II CCD diffractometer using graphite monochromated Mo-K_α_ radiation (*λ* = 0.71073 Å). Structures were solved using ShelXT direct methods or intrinsic phasing and refined using a full-matrix least-square refinement on |F|^2^ using ShelXL^39–41^. All programs were used within the Olex suites^42^. All non-hydrogen atoms were refined with anisotropic displacement parameters. H-atom parameters were constrained to parent atoms and refined using a riding model except H1 and H2, which were located in the difference Fourier maps and refined with isotropic displacement parameters. All X-ray crystal structures were analysed and illustrated using Mercury 4.1.0. X-ray data are presented in Supplementary Tables 9–14.

### NCI plots

All structures were optimised (atom-only) using CASTEP17.21^43^, with on-the-fly pseudopotentials and a plane-wave energy cut-off of 750 eV, coupled to the PBE DFT functional and TS dispersion correction scheme^44–46^. Brillouin zone sampling was 0.05 Å^−1^. Geometry convergence criteria: energy tolerance = 2 × 10^−5^ eV atom^−1^, max force = 0.05 eV Å^−1^, max atomic displacement = 2 × 10^−3^ Å. Following geometry optimisation, charge density cube files were generated using the CASTEP2CUBE utility, and subsequently used to generate non-covalent interaction (NCI) plots using the CRITIC2 code^47–49^. The graphical output from CRITIC2 was processed using VMD1.9.3 ^50^ and Origin2019.

### Powder X-ray diffraction

Powder X-ray Diffraction (PXRD) data were collected on a sample of [HL][AuCl_4_] precipitated by the addition of L (5.9 mg, 0.02 mmol) to a solution of HAuCl_4_ (0.01 M) in HCl (6 M) using a Bruker D2 phaser diffractometer in reflection geometry with Cu Kα radiation (*λ* = 1.541 Å). A LynxEye position sensitive detector was used to collect data over the 2θ range 6–65° for 15 min. Sample preparation involved grinding powder samples, mixing with acetone, and depositing a thin layer on a zero-background silicon (911) substrate. The data were analysed using a Pawley fitting routine in the Topas Academic (version 6) software suite.

## Supplementary information

Supplementary Information

Description of Additional Supplementary Files

Supplementary Movie 1

## Data Availability

X-ray data are available free of charge from the Cambridge Crystallographic Data Centre (https://www.ccdc.cam.ac.uk/data_request/cif) under reference numbers CCDC-2084239 ([HL][AuCl_4_]), CCDC-2084236 ([HL][AuBr_4_]), CCDC-2084238 ([HL][FeCl_4_]), CCDC-2084235 ([HL]_2_[SnCl_6_](H_2_O)), CCDC-2084240 ([HL]_2_[PtCl_6_](H_2_O)), CCDC-2084241 [HL][(H_3_O)(H_2_O)_2_][CoCl_4_], and CCDC-2084237 [HL][H_3_O(H_2_O)_2_][ZnCl_4_]. The quantitative metal analyses and NMR data are available in the Edinburgh DataShare repository using the identifier 10.7488/ds/3115^51^. The authors declare that all other data supporting the findings of this study are available within the paper and its supplementary information files.
