# Supplementary material for: Tuneable separation of gold by selective precipitation using a simple and recyclable diamide
Source: Nat Commun. 2021 Oct 29;12:6258. doi: 10.1038/s41467-021-26563-7 (PMC8556376; doi:10.1038/s41467-021-26563-7)
Supplement: Supplementary file 1 — Supplementary Information [file 41467_2021_26563_MOESM1_ESM.pdf]

Supplementary Information

Tuneable separation of gold by selective precipitation using a simple and recyclable diamide.

Luke M. M. Kinsman,[1] Bryne T. Ngwenya,[2] Carole A. Morrison,[1] Jason B. Love[1]*

[1]EaStCHEM School of Chemistry, University of Edinburgh, Edinburgh EH9 3FJ, UK; [2]School of Geosciences, University of Edinburgh, Edinburgh EH9 3FE, UK.

Supplementary Table 1. Precipitation experiments with Au dissolved in various aqueous matrices. [a]Conditions: 2 mL Au solution contacted with 0.059 g L for 24 hours, room temperature. Solution filtered and diluted 100 x or 1000 x in 2% HNO_3 prior to ICP-MS analysis. [b]Solution prepared from a 1000 ppm Au ICP-OES calibration standard solution. [c]Conditions: 2 mL Au solution contacted with 0.059 g L for 1 hour, room temperature. Solution filtered and diluted 100 x in 2% HNO_3 prior to ICP-OES analysis. [d]Au^0 added to sulfuric acid solution with a few drops of 30% hydrogen peroxide added to aid dissolution of Au. All experiments performed in triplicate.

Aqueous matrix	Initial Au concentration (mg L^{-1})	Au concentration after contact with L (mg L^{-1})	% Precipitated
10 ppm Au in 2 M HCl [a,b]	9.24	1.01	89.0
10 ppm Au in 6 M HCl [a,b]	9.44	1.51	84.0
0.01 M $HAuCl_4$ in 2M HCl [a]	2536	5.88	99.8
0.01 M $HAuCl_4$ in 6 M HCl [a]	2228	2.32	99.9
0.01 M $HAuCl_4$ in 100 % Aqua regia [c]	1920	6.00	99.7
0.01 M $HAuCl_4$ in 20 % Aqua regia [c]	1920	4.00	99.8
2 M H_2SO_4 and 2 M NaCl [c,d]	1940	12.2	99.4
2 M H_2SO_4 and 2 M NaBr [c.d]	1930	9.00	99.5

Supplementary Table 2. Precipitation of $HAuCl_4$ by L from 2 M HCl followed by its release using deionised water. All solutions were diluted 100x prior to ICP-OES analysis.

Sample	Au concentration (mg L^{-1})	% Precipitation	% Stripping (cumulative)
0.01 M $HAuCl_4$ in 2 M HCl (feed solution)	1940	-	
2 M HCl solution after contact with L	8.00	99.6 %	
Deionised water after contact with [HL][$AuCl_4$] solids (2 mL) 1st wash	103	-	5.3
Deionised water after contact with [HL][$AuCl_4$] solids (2 mL) 2nd wash	605	-	36.6
Deionised water after contact with [HL][$AuCl_4$] solids (2 mL) 3rd wash	826	-	79.3
Deionised water after contact with [HL][$AuCl_4$] solids (2 mL) 4th wash	147	-	86.9
Deionised water after contact with [HL][$AuCl_4$] solids (2 mL) 5th wash	139	-	94.1

Supplementary Table 3. Cycling of L through three load/strip phases.

Cycle	1	2	3
% Au precipitated	97.7%	90.7 %	87.3 %

Supplementary Table 4. Isolation and analysis of metallic gold from mixed-metal precipitation process.

Mass of Au (mg)	Au concentration in solution (mg /L)	Mass of Au in solution (mg)	% purity of Au
1.7	5.50	1.65	97.04

Supplementary Figure 1. Selectivity for gold in the presence of 28 other elements from ICP-MS standard solutions. * The negative adsorption efficiencies of some of the metals above are considered to be due to contamination of the samples from elements commonly present in water and on the experimental tools. Error bars are the standard deviation of three replicate experiments.

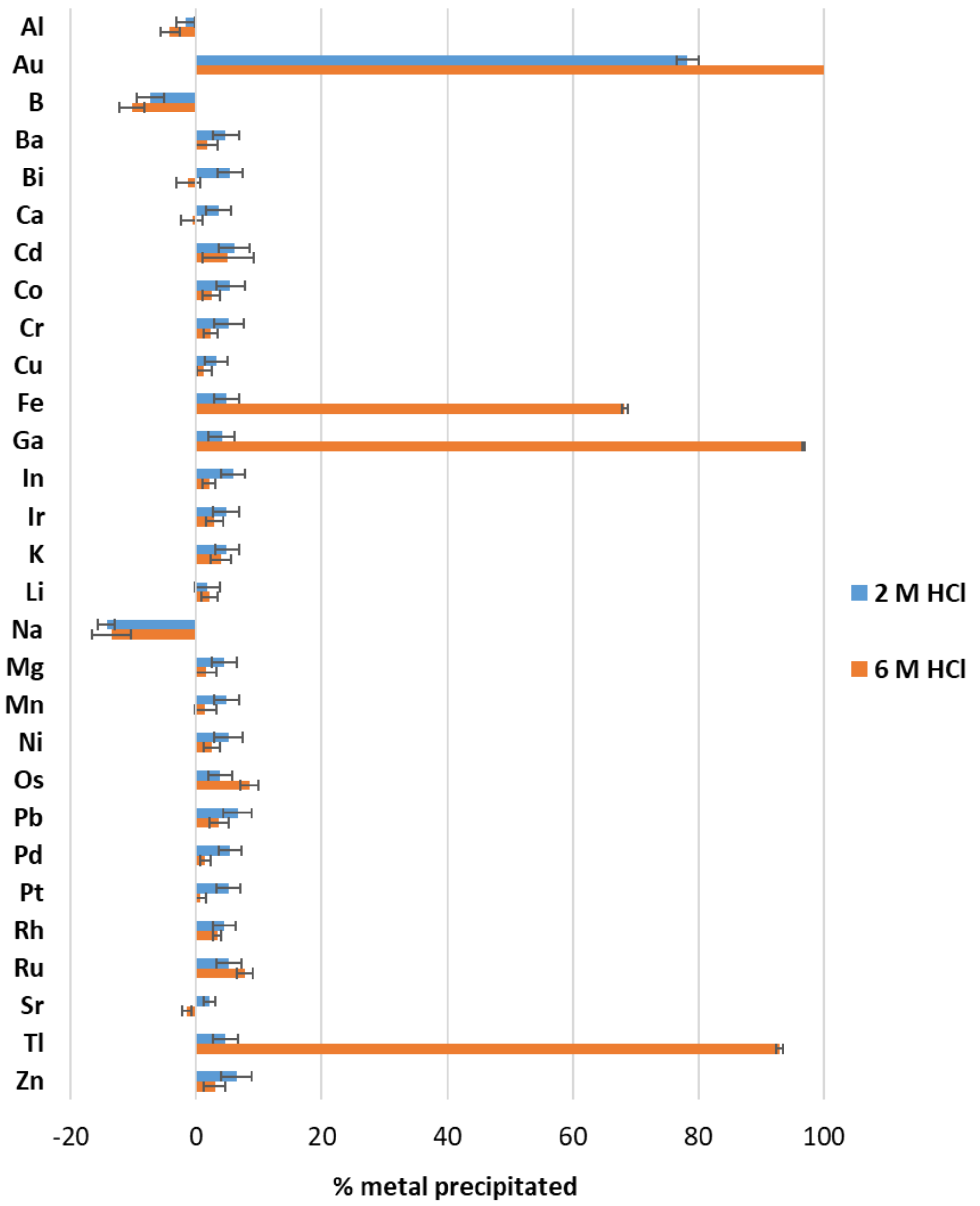

Supplementary Table 5. Precipitation of $HAuCl_4$ from a 20% aqua regia mixed-metal solution derived directly from waste printed circuit boards. See also Supplementary Figure 2.

Sample	Au (mg/L)	Cu (mg/L)	Ni (mg/L)	Pb (mg/L)	Sn (mg/L)
Feed solution	180	15400	105	818	1650
Feed solution after contact with L	3.45	15400	108	829	1660
% metal precipitated	98.1	0	0	0	0

Supplementary Figure 2. Photographs of gold precipitation from e-waste. a Photograph of e-waste pieces **b** photograph of aqua-regia solution after dissolution of e-waste **c** Photograph of [HL][$AuCl_4$] precipitate from e-waste solution.

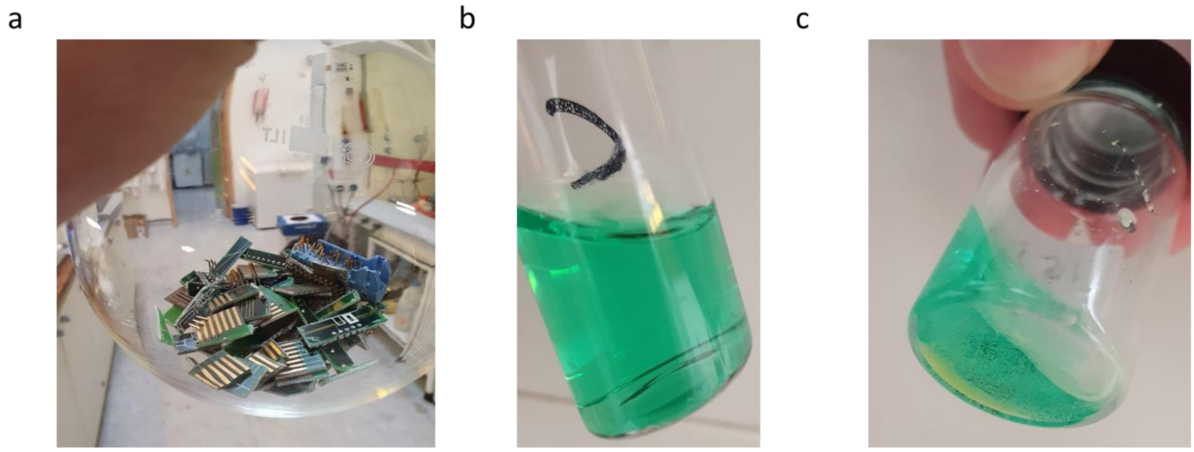

Supplementary Figure 3. Non-covalent bonding interactions. 3D isosurface plots (s = 1.0 au, -0.00025 (blue scale) < ρ < 0 (green scale) au) (left and centre), alongside 2D ρ *vs* s plots (right) for the crystal structures of **a** [HL][$AuCl_4$] and [HL][$FeCl_4$], **b** $[HL]_2[PtCl_6].H_2O$ and $[HL]_2[SnCl_6].H_2O$, and **c** [HL][$H_3O(H_2O)_2$][$CoCl_4$] and [HL][$H_3O(H_2O)_2$][$ZnCl_4$].

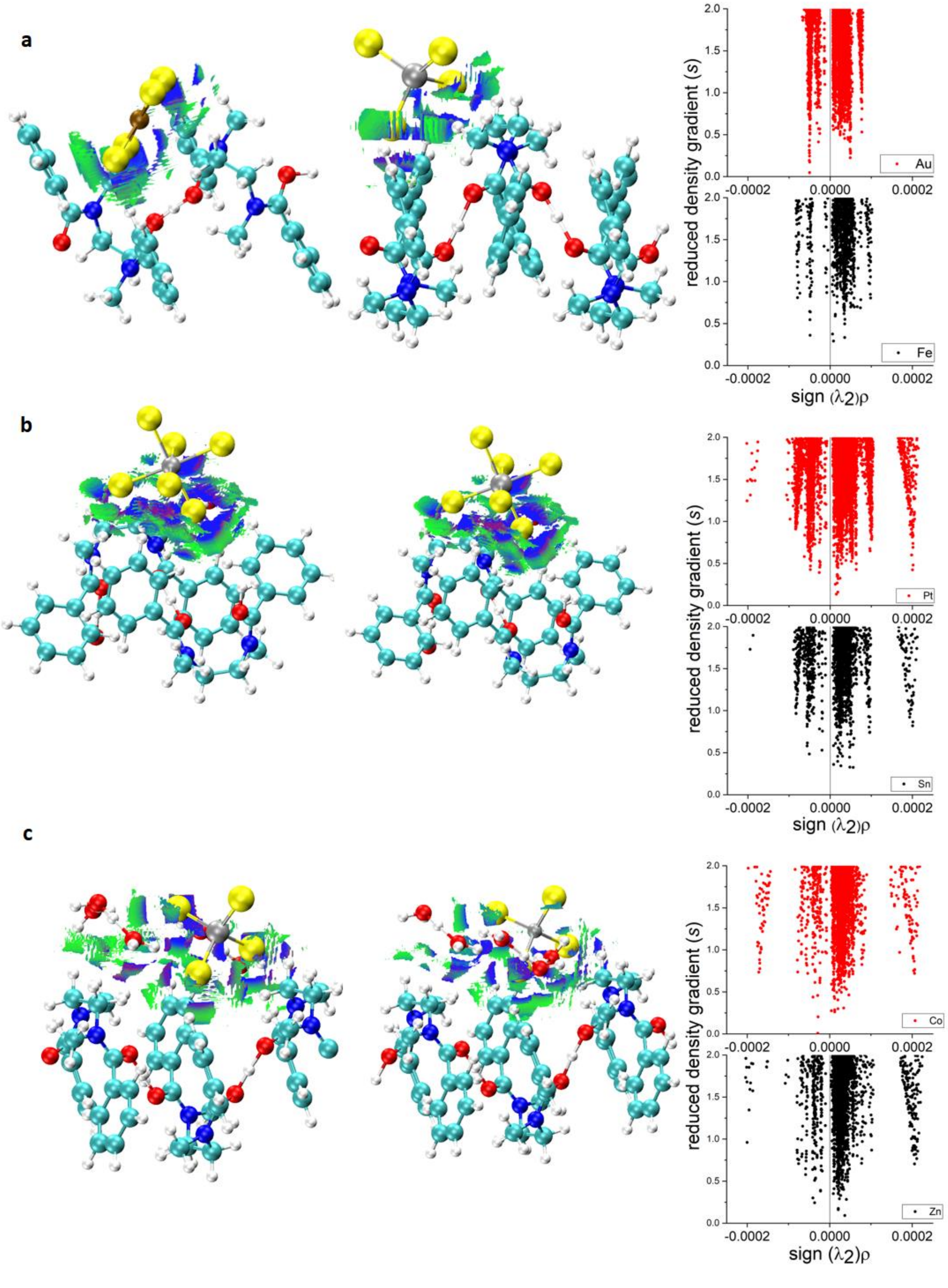

Supplementary Figure 4. X-ray crystal structures of the precipitates. Crystals formed by interaction of the diamide L with metal halides in various concentrations of HCl. The structures show how the metalates are accommodated within the intermolecular proton-chelated superstructure formed upon protonation of L.

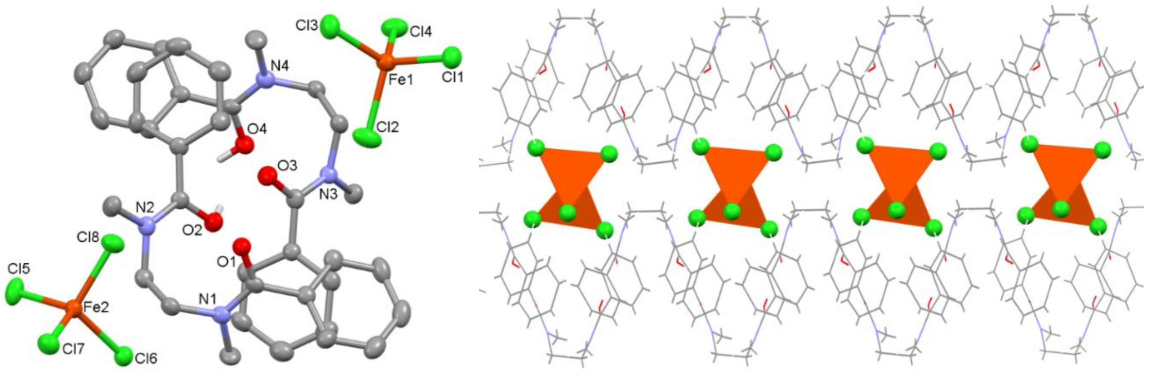

a X-ray crystal structure of [HL][$FeCl_4$]. For clarity, all hydrogens except those involved in hydrogen bonding are omitted (displacement ellipsoids are drawn at 50% probability, two molecules in the asymmetric unit).

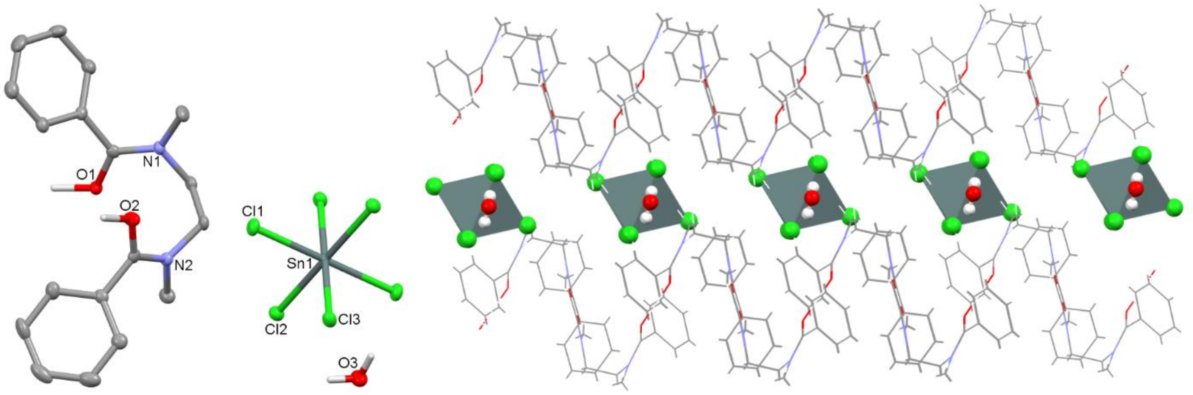

b X-ray crystal structure of $[HL]_2[SnCl_6](H_2O)$. For clarity, only one of the two [HL] cations is shown and all hydrogens except those involved in hydrogen bonding are omitted (displacement ellipsoids are drawn at 50% probability).

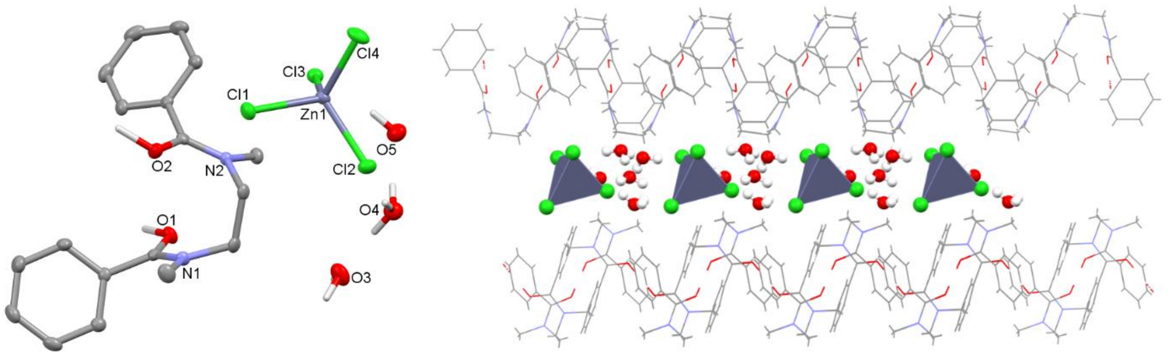

c X-ray crystal structure of $[HL][H_3O(H_2O)_2][ZnCl_4]$. For clarity, all hydrogens except those involved in hydrogen bonding are omitted (displacement ellipsoids are drawn at 50% probability).

Supplementary Figure 5. Percentage of gold precipitated from 2, 4 or 6 M HCl solutions of 0.01 M $HAuCl_4$ over time. Conditions: 0.02 mmol L stirred at 500 rpm with 2 mL $HAuCl_4$ in 2, 4 or 6 M HCl at 20 °C.

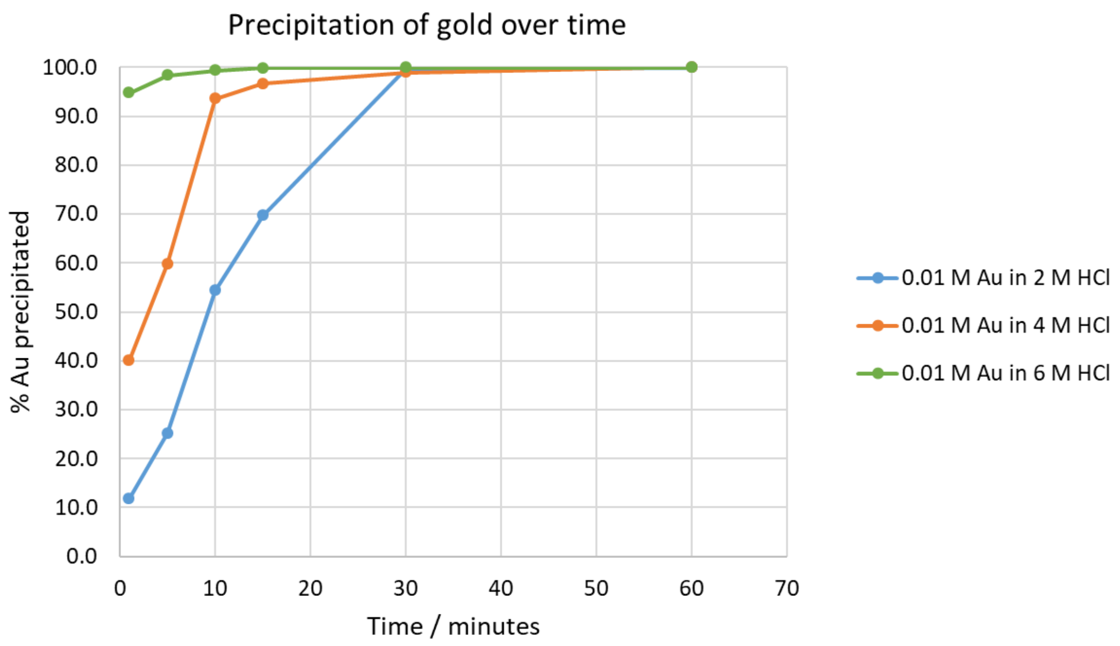

Supplementary Figure 6. Percentage of gold precipitated from 0-2 M HCl solutions of 0.01 M $HAuCl_4$. Conditions: 0.02 mmol L stirred at 500 rpm with 2 mL $HAuCl_4$ in 0-2 M HCl solutions for 1 h at 20 °C.

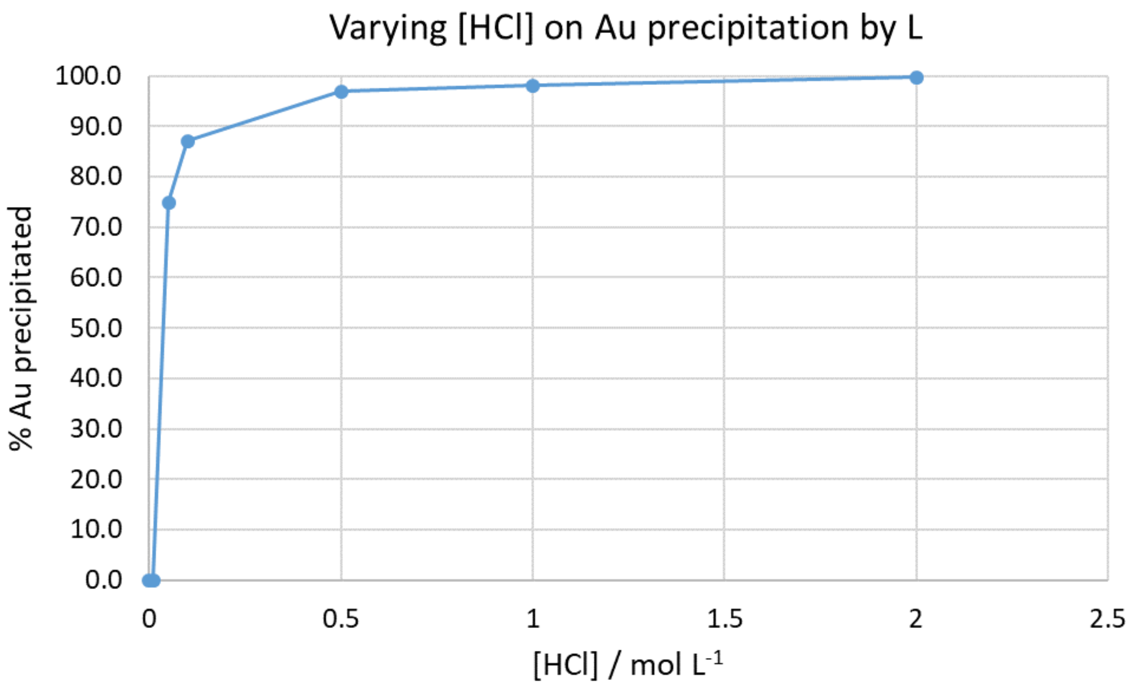

Supplementary Figure 7. Stacked ^{1}H NMR spectra (400 MHz) of L dissolved in 2 M HCl/D_2O.

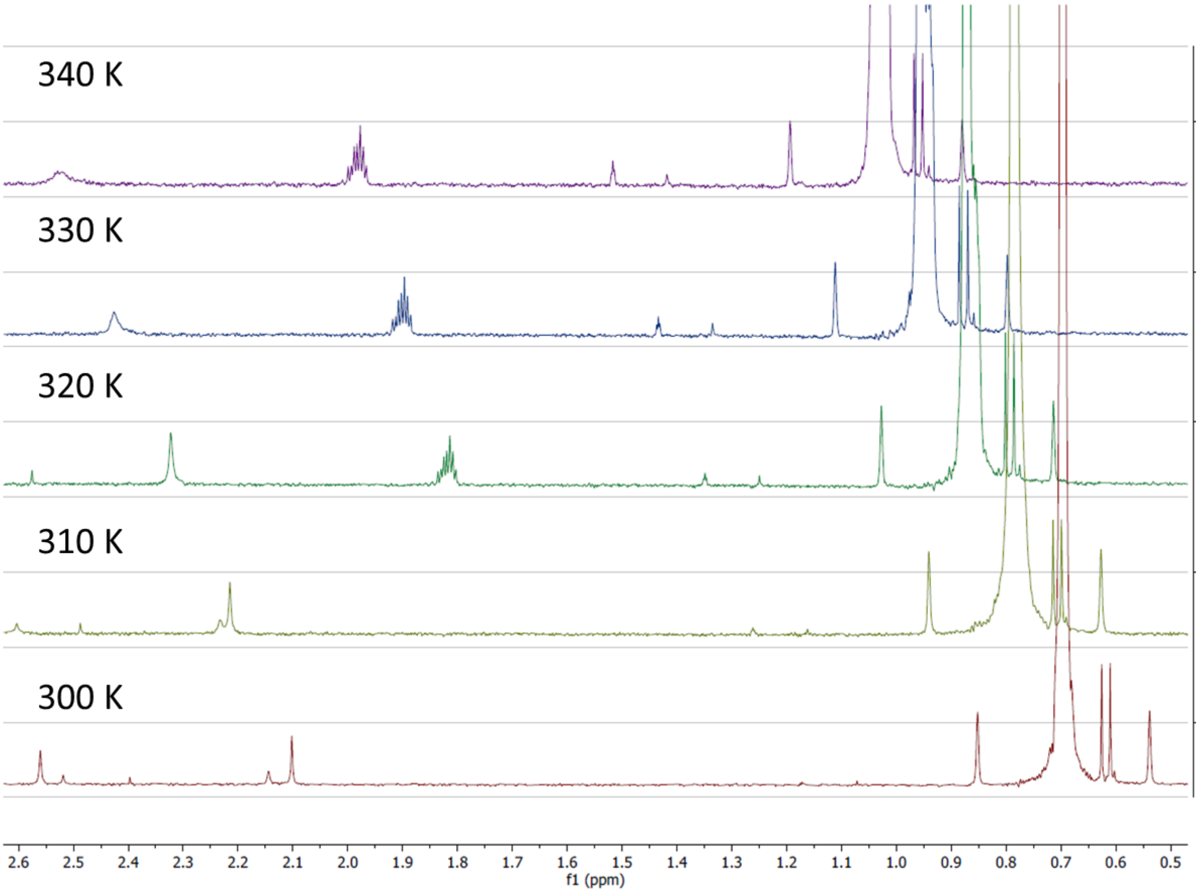

Supplementary Table 6. NMR calculations for the quantification of L in 2 M HCl/D_2O solution.

Internal standard in NMR tube = 5.04 x10^{-5} mol

Temperature / K	Integral of NCH_3 signal at 2.1 ppm	No. of NCH_3 protons	Internal standard integral	No. of internal standard protons	$R_{L/IS}$	Concentration of L / mM
300	1	6	208.89	9	0.0072	0.60
310	1	6	156.52	9	0.0096	0.80
320	1	6	158.35	9	0.0095	0.79
330	1	6	171.89	9	0.0087	0.73
340	1	6	156.23	9	0.0096	0.80

Supplementary Figure 8. Stacked ^1H NMR spectra (400 MHz) of L dissolved in 6 M HCl/D_2O.

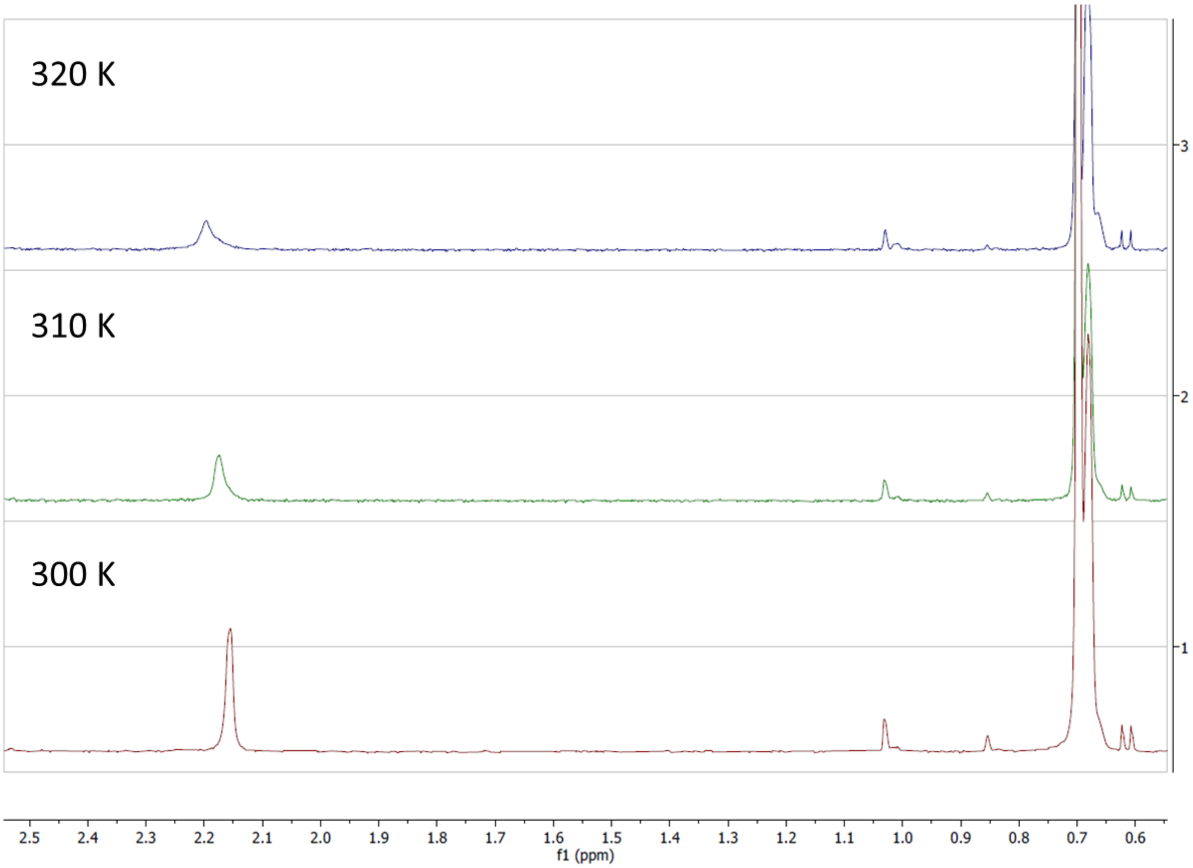

Note that 310 K and 320 K spectra in this figure are both magnified 8x. Additionally, due to difficulties with sample tuning and locking, these spectra were manually referenced to the t-BuOH internal standard after acquiring the data to maintain consistency with the 2 M HCl dataset above.

Supplementary Table 7. NMR calculations for the quantification of L in 6 M HCl/D_2O solution.

Internal standard in NMR tube = 5.04 x10^{-5} mol

Temperature / K	Integral of NCH_3 signal at 2.1 ppm	No. of NCH_3 protons	Internal standard integral	No. of internal standard protons	$R_{L/IS}$	Concentration of L / mM
300	1	6	14.46	9	0.104	8.71
310	1	6	13.76	9	0.109	9.15
320	1	6	10.84	9	0.138	11.61

Supplementary Figure 9. Percentage of gold precipitated after 5 minutes from 2 M HCl solutions of 0.005 M $HAuCl_4$ at varying temperatures. Conditions: 0.02 mmol L stirred at 500 rpm with 2 mL $HAuCl_4$ in 2 M HCl solutions for 5 minutes at 20, 40, and 80 °C.

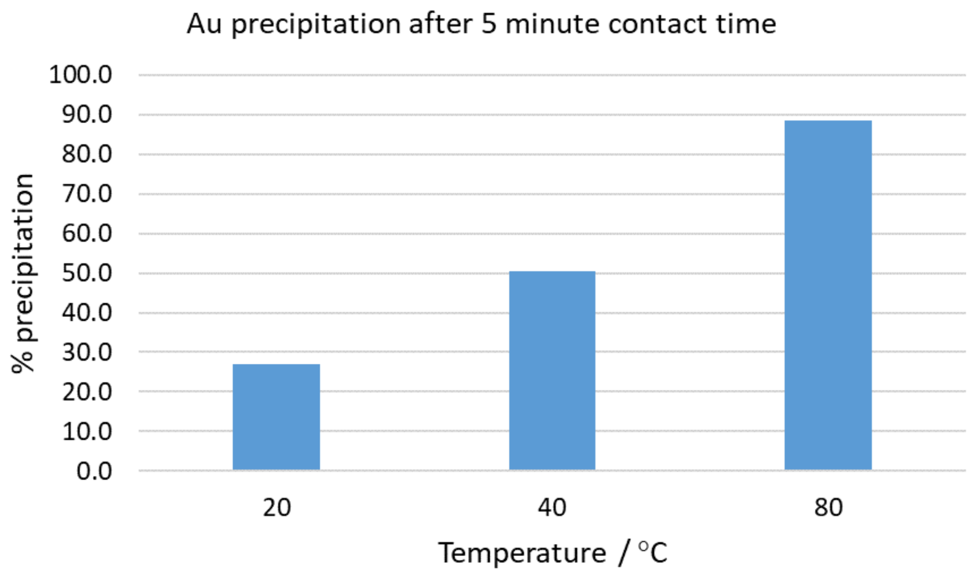

Supplementary Figure 10. Powder X-ray diffraction. [HL][$AuCl_4$] precipitate from a mixture of L (5.9 mg, 0.02 mmol) and $HAuCl_4$ (0.01 M) in 6 M HCl showing the observed data (green), the calculated profile from a multiphase Pawley refinement of [HL][$AuCl_4$] and L (red), and the difference profile (grey). Blue tick marks are associated with [HL][$AuCl_4$] whereas green tick marks are associated with L.

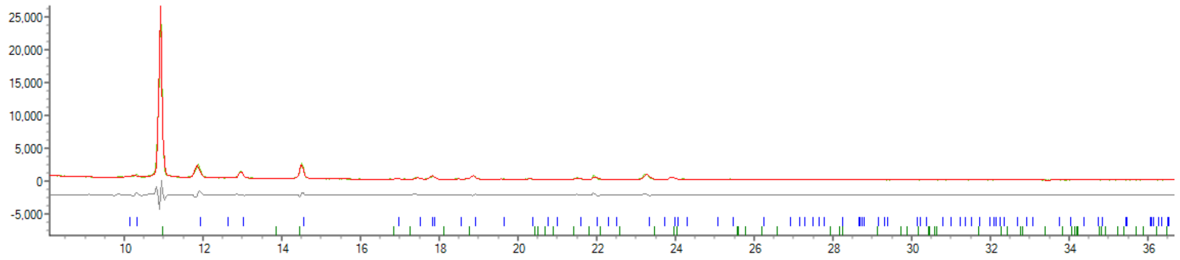

Supplementary Figure 11. Photograph of H-tube apparatus for selective stripping experiments.

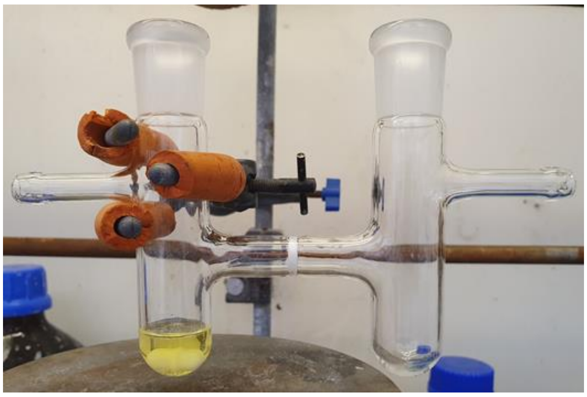

Supplementary Table 8. List of emission wavelengths used for ICP-OES analysis.

Metal	Wavelength / nm
Al	**396.153**
Au	**267.595**
B	**249.772**
Ba	**233.527**
Bi	**306.766**
Ca	**317.933**
Cd	**228.802**
Co	**228.616**
Cr	**205.56**
Cu	**327.393**
Fe	**238.204**
Ga	**417.206**
In	**325.609**
Ir	**224.268**
K	**766.49**
Li	**610.362**
Na	**589.59**
Mg	**285.213**
Mn	**257.61**
Ni	**221.648**
Os	**225.585**
Pb	**220.353**
Pd	**340.458**
Pt	**265.945**
Rh	**343.489**
Ru	**240.272**
Sr	**460.733**
Tl	**351.924**
Zn	**213.857**

Supplementary Table 9. X-ray data for [HL][$AuCl_4$]

Chemical Formula	$AuCl_4C_{18}H_{21}N_2O_2$
M_r	636.13
Crystal system	Monoclinic
Space group	I2/a
Temperature (K)	120
a/Å	12.0848 (7)
b/Å	16.9574 (2)
c/Å	16.5644 (10)
α/°	90
β/°	142.074 (13)
γ/°	90
Volume/Å	2086.4
Z	4
Radiation type	Mo K_α
μ/mm^{-1}	7.58
Diffractometer	Rigaku Oxford Diffraction XCalibur
Absorption correction	Multi-scan<br>CrysAlis PRO 1.171.40.84a (Rigaku Oxford Diffraction, 2020) Empirical absorption correction using spherical harmonics, implemented in SCALE3 ABSPACK scaling algorithm.
T_{min}, T_{max}	0.610, 1.000
No. of measured, independent and observed [I > 2σ(I)] reflections	22821, 2669, 2397
R_{int}	0.028
$(\sin \theta/\lambda)_{max}$ (Å^{-1})	0.692
$R[F^2 > 2\sigma(F^2)]$, $wR(F^2)$, S	0.016, 0.030, 1.07
No. of reflections	2669
No. of parameters	129
H-atom treatment	H atoms treated by a mixture of independent and constrained refinement
$\Delta\rho_{max}$, $\Delta\rho_{min}$ (e Å^{-3})	0.53, −0.52
CCDC number	2084239

Supplementary Table 10. X-ray data for [HL][$FeCl_4$]

Chemical Formula	$FeCl_4C_{18}H_{21}N_2O_2$
M_r	495.02
Crystal system	Monoclinic
Space group	$P2_1$
Temperature (K)	120
a/Å	8.6282 (1)
b/Å	19.0047 (3)
c/Å	13.8701 (3)
α/°	90
β/°	105.557 (2)
γ/°	90
Volume/Å	2191.04 (7)
Z	4
Radiation type	Cu K_α
μ/mm^{-1}	10.14
Diffractometer	Rigaku Oxford Diffraction SuperNova
Absorption correction	Multi-scan<br>SADABS2016/2 (Bruker,2016/2) was used for absorption correction. wR2(int) was 0.1221 before and 0.0887 after correction. The Ratio of minimum to maximum transmission is 0.6375. The λ/2 correction factor is Not present.
T_{min}, T_{max}	0.481, 0.754
No. of measured, independent and observed [I > 2σ(I)] reflections	44934, 9025, 8375
R_{int}	0.087
$(\sin \theta/\lambda)_{max}$ (Å^{-1})	0.629
$R[F^2 > 2\sigma(F^2)]$, $wR(F^2)$, S	0.055, 0.143, 1.06
No. of reflections	9025
No. of parameters	500
H-atom treatment	H atoms treated by a mixture of independent and constrained refinement
$\Delta\rho_{max}$, $\Delta\rho_{min}$ (e Å^{-3})	0.44, -0.61
Absolute structure	Refined as an inversion twin
Absolute structure parameter	0.475 (8)
CCDC number	2084238

Supplementary Table 11. X-ray data for $[HL]_2[SnCl_6](H_2O)$

Chemical Formula	$SnCl_6C_{36}H_{42}N_4O_4.1.265(H_2O)$
M_r	948.83
Crystal system	Triclinic
Space group	P-1
Temperature (K)	120
a/Å	8.7720 (4)
b/Å	11.1752 (5)
c/Å	12.0751 (6)
α/°	113.632 (4)
β/°	103.282 (4)
γ/°	97.177 (4)
Volume/Å	1023.69 (9)
Z	1
Radiation type	Mo K_{α}
μ/mm^{-1}	1.06
Diffractometer	Rigaku Oxford Diffraction SuperNova
Absorption correction	Multi-scan<br>*CrysAlis PRO* 1.171.40.84a (Rigaku Oxford Diffraction, 2020) Empirical absorption correction using spherical harmonics, implemented in SCALE3 ABSPACK scaling algorithm.
T_{min}, T_{max}	0.533, 1.00
No. of measured, independent and observed [I > 2σ(I)] reflections	18433, 5179, 4332
R_{int}	0.063
$(\sin \theta/\lambda)_{max}$ ($Å^{-1}$)	0.699
$R[F^2 > 2\sigma(F^2)]$, $wR(F^2)$, S	0.041, 0.070, 1.05
No. of reflections	5179
No. of parameters	258
H-atom treatment	H atoms treated by a mixture of independent and constrained refinement
$\Delta\rho_{max}$, $\Delta\rho_{min}$ (e $Å^{-3}$)	0.70, -0.59
CCDC number	2084235

Supplementary Table 12. X-ray data for $[HL]_2[PtCl_6](H_2O)$

Chemical Formula	$PtCl_6C_{36}H_{42}N_4O_4.2(H_2O)$
M_r	1038.56
Crystal system	Triclinic
Space group	P-1
Temperature (K)	100
a/Å	8.7579 (10)
b/Å	11.0954 (14)
c/Å	12.0668 (15)
α/°	113.978 (5)
β/°	103.941 (5)
γ/°	96.913 (5)
Volume/Å	1007.8 (2)
Z	1
Radiation type	Mo K_α
μ/mm^{-1}	3.93
Diffractometer	Bruker D8 Venture
Absorption correction	Multi-scan<br>SADABS2016/2 (Bruker,2016/2) was used for absorption correction. wR2(int) was 0.1037 before and 0.0540 after correction. The Ratio of minimum to maximum transmission is 0.6929. The λ/2 correction factor is Not present.
T_{min}, T_{max}	0.518, 0.748
No. of measured, independent and observed [I > 2σ(I)] reflections	91497, 12086, 11750
R_{int}	0.045
$(\sin \theta/\lambda)_{max}$ (Å^{-1})	0.910
$R[F^2 > 2\sigma(F^2)]$, $wR(F^2)$, S	0.025, 0.051, 1.06
No. of reflections	12086
No. of parameters	259
H-atom treatment	H atoms treated by a mixture of independent and constrained refinement
$\Delta\rho_{max}$, $\Delta\rho_{min}$ (e Å^{-3})	1.07, -2.74
CCDC number	2084240

Supplementary Table 13. X-ray data for [HL][$H_3O(H_2O)_2$][$CoCl_4$]

Chemical Formula	$Cl_4CoC_{18}H_{21}N_2O_2{\cdot}2(H_2O){\cdot}H_3O$
M_r	553.15
Crystal system	Monoclinic
Space group	P21/n
Temperature (K)	120
a/Å	9.0978 (2)
b/Å	11.4600 (3)
c/Å	23.7020 (7)
α/°	90
β/°	100.514 (2)
γ/°	90
Volume/Å	2429.70 (11)
Z	4
Radiation type	Mo K_α
μ/mm^{-1}	1.18
Diffractometer	Rigaku Oxford Diffraction XCalibur
Absorption correction	Multi-scan<br>CrysAlis PRO 1.171.41.99a (Rigaku Oxford Diffraction, 2021) Empirical absorption correction using spherical harmonics, implemented in SCALE3 ABSPACK scaling algorithm.
T_{min}, T_{max}	0.908, 1.000
No. of measured, independent and observed [I > 2σ(I)] reflections	33219, 6819, 5626
R_{int}	0.044
$(\sin \theta/\lambda)_{max}$ ($Å^{-1}$)	0.722
$R[F^2 > 2\sigma(F^2)]$, $wR(F^2)$, S	0.037, 0.070, 1.03
No. of reflections	6819
No. of parameters	306
H-atom treatment	H atoms treated by a mixture of independent and constrained refinement
$\Delta\rho_{max}$, $\Delta\rho_{min}$ (e $Å^{-3}$)	0.41, -0.36
CCDC number	2084241

Supplementary Table 14. X-ray data for [HL][$H_3O(H_2O)_2$][$ZnCl_4$]

Chemical Formula	$Cl_4ZnC_{18}H_{21}N_2O_2 \cdot 2(H_2O) \cdot H_3O$
M_r	559.59
Crystal system	Monoclinic
Space group	$P2_1/n$
Temperature (K)	120
a/Å	9.1085 (2)
b/Å	11.4514 (2)
c/Å	23.7051 (5)
α/°	90
β/°	100.502 (2)
γ/°	90
Volume/Å	2431.14 (9)
Z	4
Radiation type	Mo K_α
μ/mm^{-1}	1.48
Diffractometer	Rigaku Oxford Diffraction XCalibur
Absorption correction	Multi-scan<br>CrysAlis PRO 1.171.40.53 (Rigaku Oxford Diffraction, 2019) Analytical numeric absorption correction using a multifaceted crystal model based on expressions derived by R.C. Clark & J.S. Reid. (Clark, R. C. & Reid, J. S. (1995). Acta Cryst. A51, 887-897) Empirical absorption correction using spherical harmonics, implemented in SCALE3 ABSPACK scaling algorithm.
T_{min}, T_{max}	0.811, 0.962
No. of measured, independent and observed [I > 2σ(I)] reflections	44451, 4437, 3625
R_{int}	0.073
$(\sin \theta/\lambda)_{max}$ (Å^{-1})	0.602
$R[F^2 > 2\sigma(F^2)]$, $wR(F^2)$, S	0.031, 0.066, 1.04
No. of reflections	4437
No. of parameters	288
H-atom treatment	H atoms treated by a mixture of independent and constrained refinement
$\Delta\rho_{max}$, $\Delta\rho_{min}$ (e Å^{-3})	0.34, −0.27
CCDC number	2084237

Supplementary Figure 12. UV-Vis data for varying concentrations of [HL][$AuCl_4$] in acetonitrile.

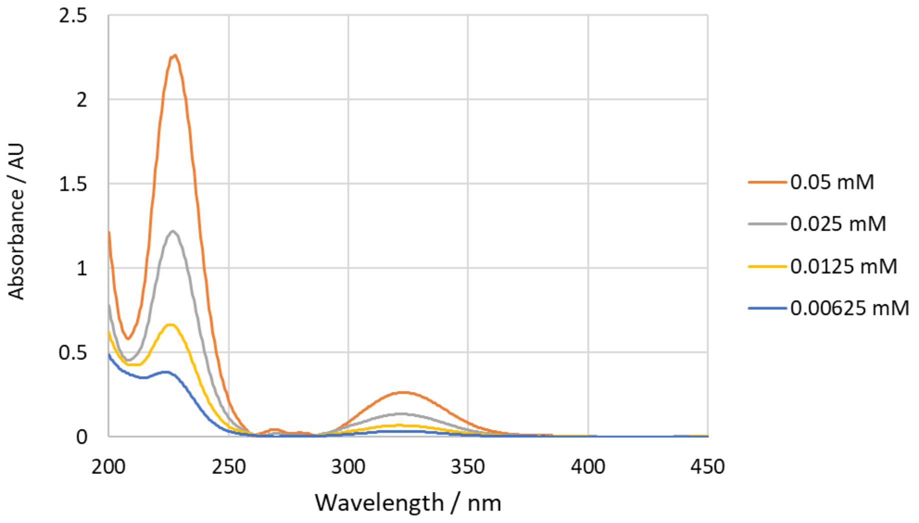

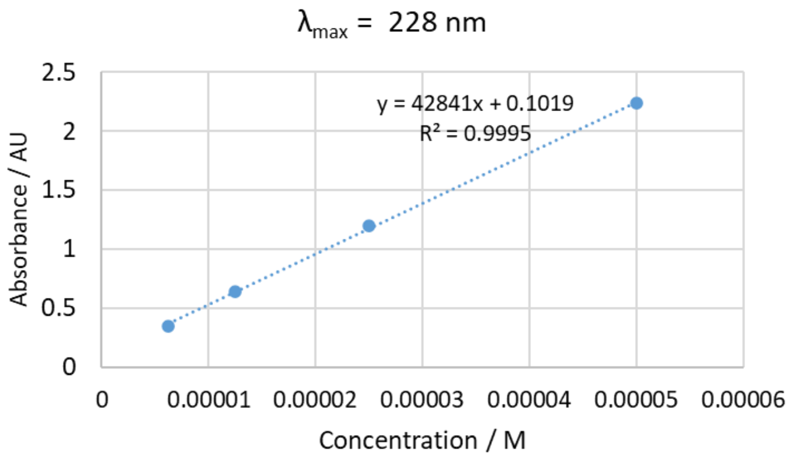

Calculation of extinction coefficient at λ_{max} 228 nm

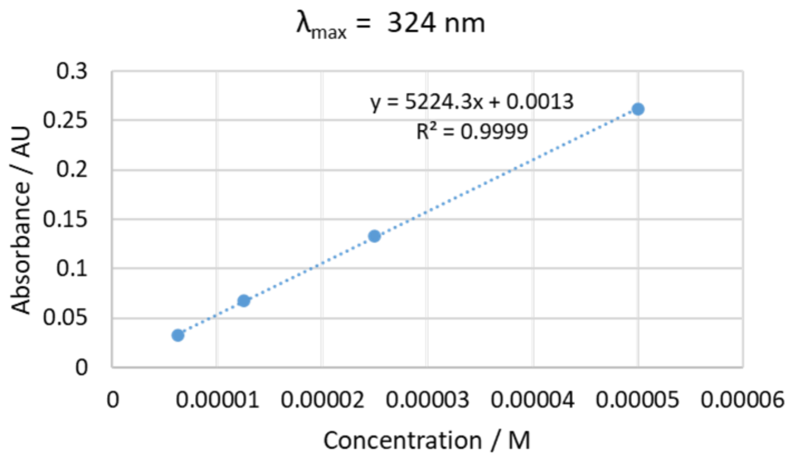

Calculation of extinction coefficient at λ_{max} 324 nm

Supplementary Figure 13. ^{1}**H NMR spectrum of L (500 MHz,** $CDCl_3$**).**

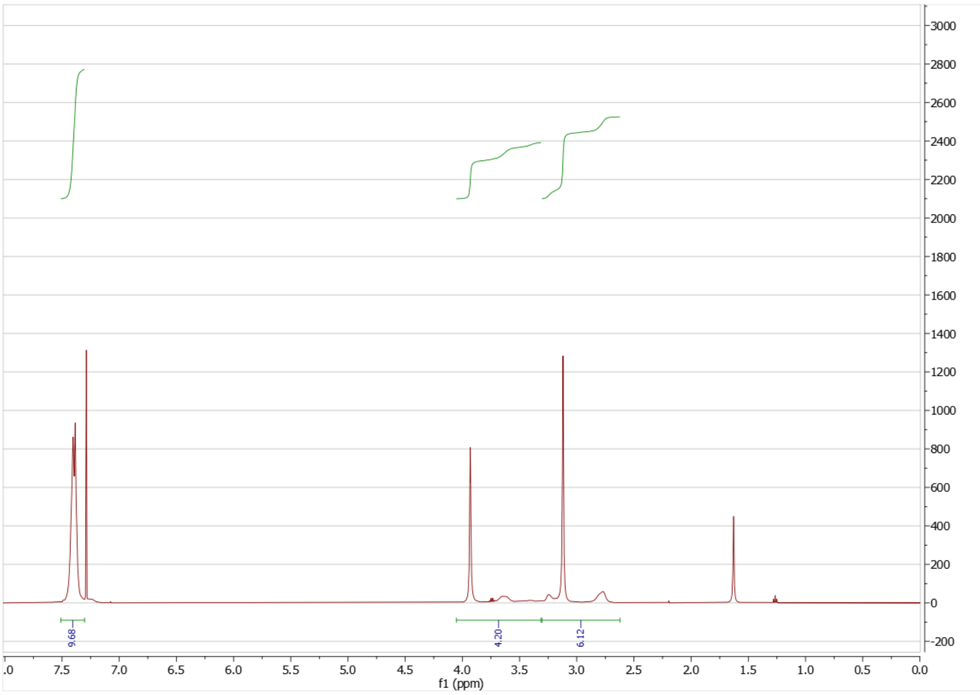

Note that three different rotational isomers are present in the ^{1}H NMR spectrum.[1,2]

Supplementary Figure 14. ^{13}**C{**^{1}**H} NMR spectrum of L (126 MHz,** $CDCl_3$**).**

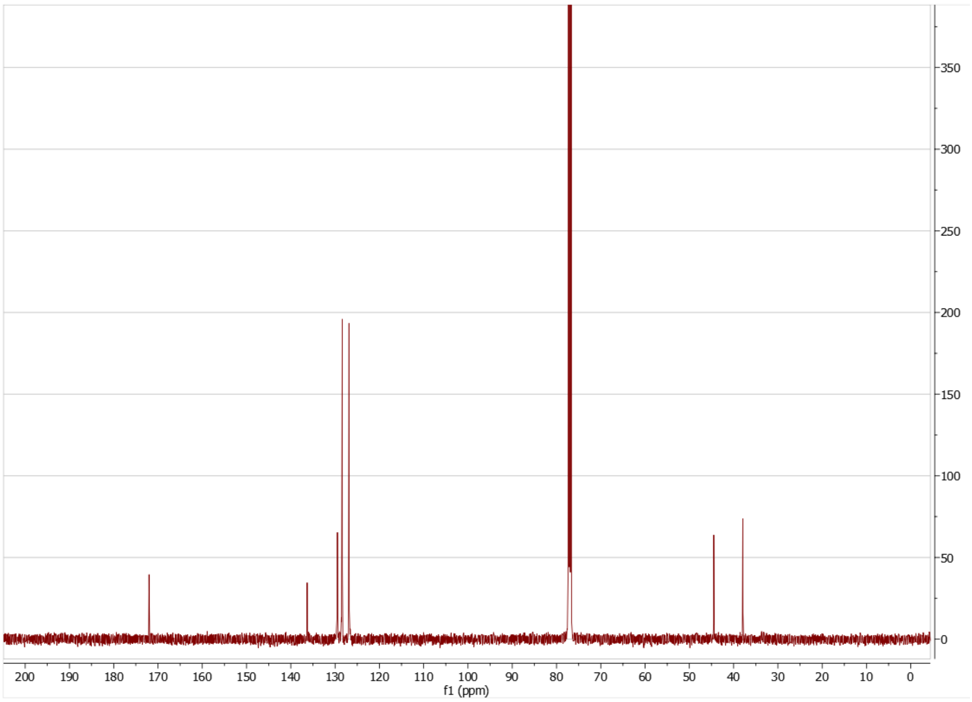

Supplementary References.

1. Kaufmann, L. *et al.* Substituent effects on axle binding in amide pseudorotaxanes: comparison of NMR titration and ITC data with DFT calculations. *Org. Biomol. Chem.* **10**, 5954-5964, (2012).
2. Cirrincione, G., Hinz, W. & Jones, R. A. Rotational isomerism of NN'-dimethyl-α,ω-bis(benzoylamino)alkanes. *J. Chem. Soc., Perkin Trans. 2*, 1089-1091, (1984).
