# Supplementary material for: Tuneable separation of gold by selective precipitation using a simple and recyclable diamide
Source: Nat Commun. 2021 Oct 29;12:6258. doi: 10.1038/s41467-021-26563-7 (PMC8556376; doi:10.1038/s41467-021-26563-7)
Supplement: Supplementary file 2 — Description of Additional Supplementary Files [file 41467_2021_26563_MOESM2_ESM.pdf]

Description of Additional Supplementary Files

Supplementary Movie 1: Movie of precipitation process
